# Supplementary material for: Highly Selective Adsorption on SiSe Monolayer and Effect of Strain Engineering: A DFT Study
Source: Sensors (Basel). 2020 Feb 12;20(4):977. doi: 10.3390/s20040977 (PMC7070421; doi:10.3390/s20040977)
Supplement: Supplementary file 1 [file sensors-20-00977-s001.pdf]

Highly selective adsorption on SiSe monolayer and effect of strain engineering: A DFT study

Quan Zhou [1], Lian Liu [1], Qipeng Liu [1], Zeping Wang [1], Chenshan Gao [1], Yufei Liu [1,2] and Huaiyu Ye [1,3,4,*]

[1] Key Laboratory of Optoelectronic Technology & Systems, Education Ministry of China, and College of Optoelectronic Engineering, Chongqing University, Chongqing 400044, China;
[2] Centre for Intelligent Sensing Technology, College of Optoelectronic Engineering, Chongqing University, Chongqing 400044, China;
[3] Shenzhen Institute of Wide-Bandgap Semiconductors, No.1088, Xueyuan Rd., Xili, Nanshan District, Shenzhen, Guangdong, China;
[4] Electronic Components, Technology and Materials, Delft University of Technology, Delft 2628 CD, the Netherlands;
*Correspondence: h.ye@tudelft.nl;

Figure S1

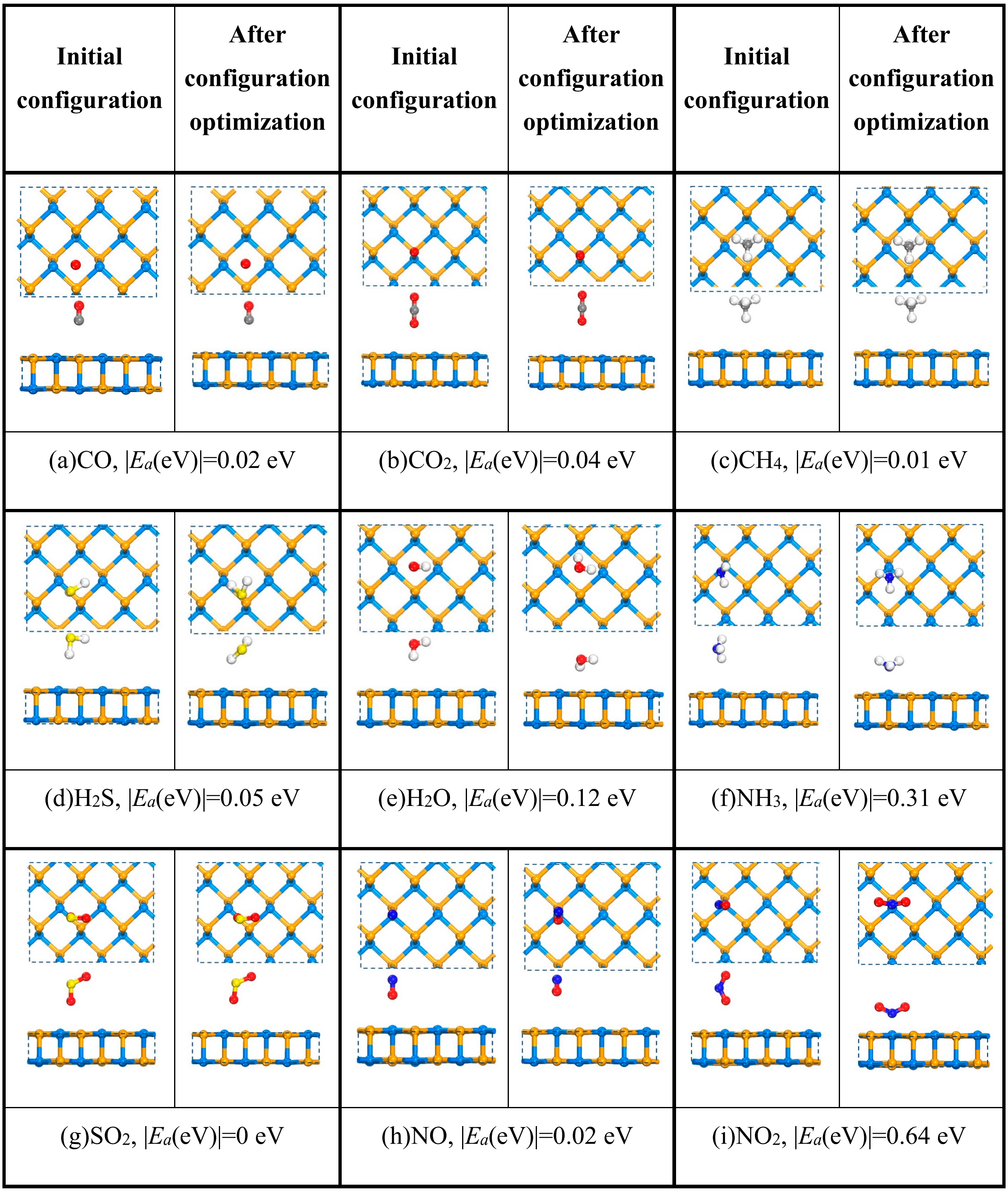

Figure S1. Front and top views of gas molecules adsorbed on a SiSe substrate in a different molecular orientation. Gas in turn are CO, CO_2, CH_4, H_2S, H_2O, NH_3, SO_2, NO and NO_2.

Figure S2

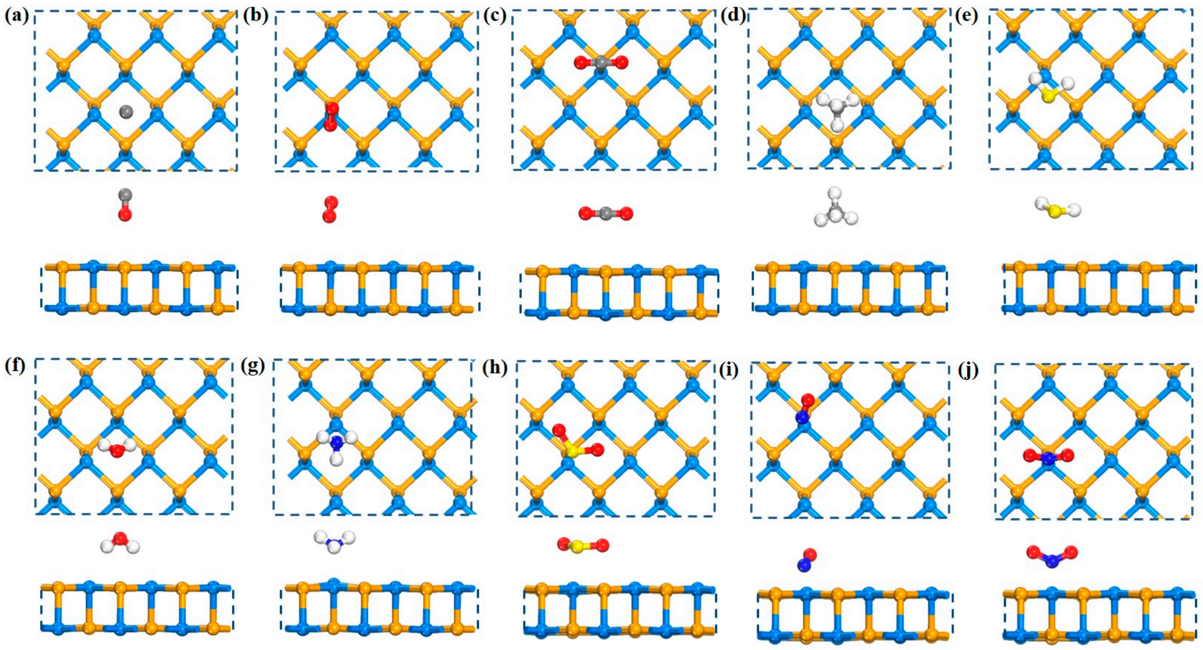

Figure S2**.** Front and top views of gas molecules adsorbed on a SiSe substrate. Gas in turn are CO, O_2, CO_2, CH_4, H_2S, H_2O, NH_3, SO_2, NO and NO_2.

Figure S3

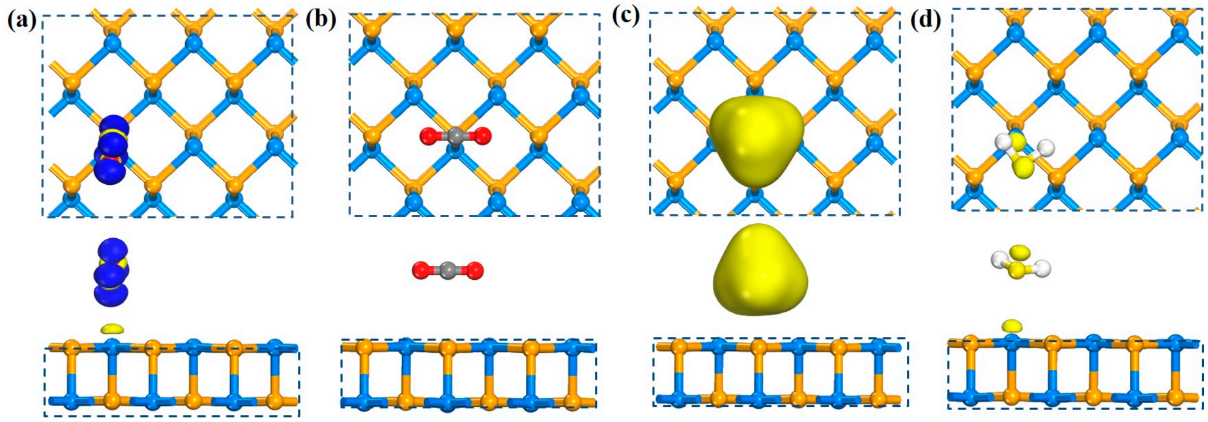

Figure S3**.** Front and top charge distribution view of charge density difference (CDD) maps, in turn are (a) O_2, (b) CO_2, (c) CH_4, (d) H_2S configuration. The isosurface is set as 0.01e/Å^3.

Figure S4

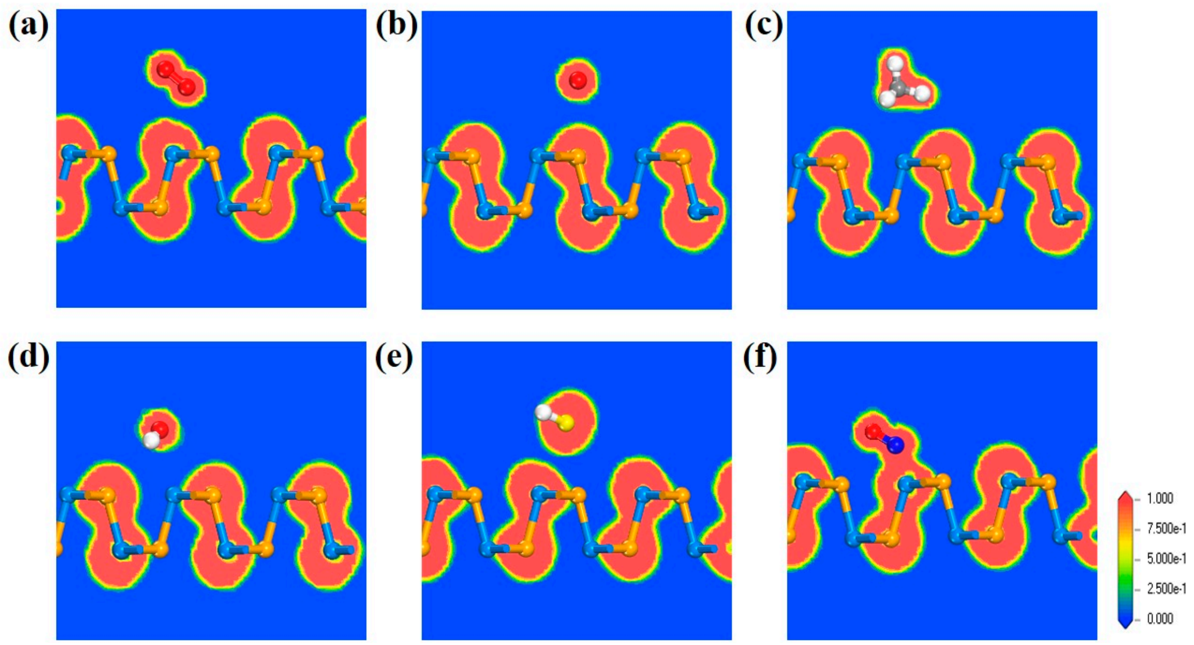

Figure S4**.** The distribution of electron localization maps of (a) O_2, (b) CO_2, (c) CH_4, (d) H_2O (e) H_2S and (f) NO configurations. The reference column for the ELF value from 0 to 1 is located on the right side of the figure. The slice of the ELF is parallel to the (100) crystal plane.

Table S1

Table S1. The adsorption energy (E_{ad}), closest distance (d) and Mulliken charge transfer (ΔQ) of strained-NH_3/SiSe configurations from X-axis, Y-axis, and biaxial directions.

Strain	E_{ax}(eV)	d_x(Å)	ΔQ_x (e)	E_{ay}(eV)	d_y(Å)	Q_y (e)	E_{axy}(eV)	d_{xy}(Å)	Q_{xy} (e)
-8%	-0.461	2.323	0.206	-0.460	2.351	0.194	-0.753	2.226	0.223
-6%	-0.430	2.37	0.191	-0.439	2.372	0.19	-0.589	2.273	0.212
-4%	-0.427	2.423	0.176	-0.420	2.392	0.185	-0.450	2.351	0.197
-2%	-0.426	2.443	0.168	-0.404	2.404	0.182	-0.418	2.384	0.188
0%	-0.414	2.471	0.153	-0.414	2.471	0.153	-0.414	2.471	0.153
2%	-0.412	2.492	0.143	-0.411	2.482	0.149	-0.407	2.503	0.139
4%	-0.405	2.508	0.136	-0.375	2.485	0.149	-0.401	2.518	0.134
6%	-0.401	2.52	0.124	-0.406	2.488	0.146	-0.400	2.497	0.122
8%	-0.396	2.526	0.119	-0.405	2.479	0.147	-0.404	2.465	0.121

Table S2

Table S2. The configuration structures of NH_3/SiSe, SO_2/SiSe, NH_3-SO_2/SiSe, SO_2-NH_3/SiSe and SO_2&NH_3/SiSe with adsorption energy (E_{ad}), Mulliken charge transfer (ΔQ) and closest distance (d).

**configuration**	E_{ad} **(eV)**	ΔQ**(SO_2)**	ΔQ**(SO_2)**	d**(NH_3)**	d**(SO_2)**
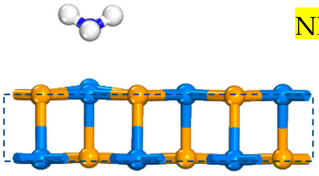 NH_3/SiSe	-0.414	0.178	\	2.471	\
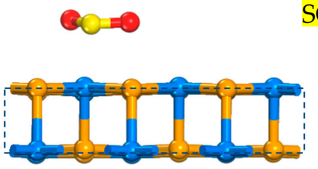 SO_2/SiSe	-0.489	\	-0.197	\	2.686
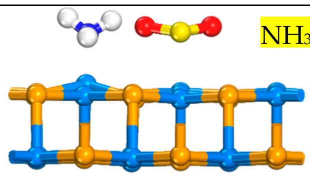 NH_3-SO_2/SiSe	-0.752	0.243	-0.278	2.226	2.639
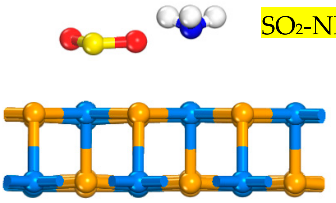 SO_2-NH_3/SiSe	-0.465	0.196	-0.220	2.431	2.760
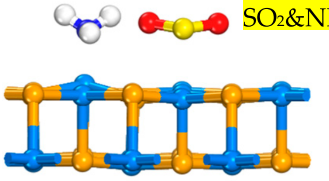 SO_2&NH_3/SiSe	-1.161	0.241	-0.276	2.278	2.646
